# The effects of post-exercise whey vs. casein protein ingestion on muscular strength, muscular endurance, and body composition in older women (50-70 years of age)

**DOI:** 10.1186/1550-2783-8-S1-P27

**Published:** 2011-11-07

**Authors:** Stacie L Urbina, Andrew White, Josh Shaw, Colin Wilborn, Brian Brabham

**Affiliations:** 1 Department of Exercise and Sport Science. University of Mary Hardin-Baylor, Belton, TX 76513, USA

## Background

As humans age, there is a measurable loss of muscle mass that occurs. Termed sarcopenia, this condition not only results in a loss of muscle mass, but also results in a loss of muscular strength and endurance (Bales, 2002). Research has shown that resistance training decreases this loss of muscle mass and muscular strength (Doherty, 2003). However, in older populations, little evidence exists in regards to the addition of whey or casein protein and the effects of each when combined with resistance training. Therefore, the purpose of this study was to examine the effects of whey versus casein protein supplementationcombined with resistance training on muscular strength, muscular endurance and body composition in older females.

## Methods

Nineteen non-resistance trained females (57.42±5.32 yrs, 163.53±6.42 cm, 56.6±9.47 kg) were matched according to bodyweight and total weight lifted and then randomized in a double blind manner to receive either whey (n=10) or casein protein (n=9).Participants ingested either casein protein (24g/d) or whey protein (24g/d) 30 minutes to 1 hour post-exercisewhile participating in a high intensity resistance training program (3 sets x 10 repetitions at 75% of 1RM), 3 days per week for 8 weeks. Ingestion occurred on non-training days at approximately the same time of day. Testing sessions were completed prior to, 4 weeks and 8 weeks post resistance training and supplementation. Each testing session included body composition measurement as determined by Dual Energy X-Ray Absorptiometry (DEXA), muscle strength measurement as determined by 1 repetition maximum (RM) on leg press and chest press as well a muscular endurance measurement as determined by a repetition to failure test at 75% of 1 repetition maximum on both the leg press and chest press. Data were analyzed using repeated measures ANOVA.

## Results

A significant time effect was observed for 1RM chest press (0 weeks: 40.66kg ± 6.72kg vs. 8 weeks: 55.07kg ± 10.29 kg, p<0.05), leg press (0 weeks: 156.73kg ± 32.69kg vs. 8 weeks: 233.13kg ±42.5kg,p<0.05), leg press repetition to failure (0 weeks: 21.79 vs. 8 weeks: 13.68, p=0.014, fat mass (0 weeks: 28.19kg ± 7.05kg vs. 8 weeks: 27.39kg ± 7.09kg, p=0.015), fat free mass (0 weeks: 40.22kg ± 4.35kg vs. 8 weeks: 41.69 kg ± 4.62 kg, p<0.05) and percent body fat (0 weeks: 40.93%±5.96% vs. 8 weeks: 39.47%±5.88%). However, no significant group or group by time interactionswere observed.

## Conclusion

When combined with 8-weeks of high intensity resistance training,there is no significant difference in whey versus casein ingestion in regards to their ability to enhance body composition, muscular strength, or muscular endurance in older females.

